# Experimental Study on Different Improvement Schemes of EICP-Lignin Solidified Silt

**DOI:** 10.3390/ma16030999

**Published:** 2023-01-21

**Authors:** Yongshuai Sun, Xinyan Zhong, Jianguo Lv, Guihe Wang, Ruilin Hu

**Affiliations:** 1 College of Water Resources & Civil Engineering, China Agricultural University, Beijing 100083, China; 2 School of Engineering and Technology, China University of Geosciences, Beijing 100083, China; 3 Key Laboratory of Shale Gas and Geoengineering, Institute of Geology and Geophysics, Chinese Academy of Sciences, Beijing 100029, China

**Keywords:** EICP, silt, lignin, urease, *Sporosarcina pasteurii*, calcium carbonate, destruction form, cumulative strain, resilience modulus, maximum bearing capacity

## Abstract

In practical engineering applications, silt is prone to liquefaction and quicksand. This paper mainly studies the improvement effects of urease, lignin and their mixture on the strength and liquefaction resistance of silt. Based on the results and phenomena of an unconfined compressive strength and dynamic triaxial test, the improvement effects of the compressive strength, deformation resistance and liquefaction resistance of silt under different improvement schemes are analyzed, and the optimal values of the cement or lignin when enzyme-induced calcium carbonate precipitation (EICP) technology, lignin alone or EICP and lignin are obtained. The results show that the optimum concentration of the constant temperature and humidity sample (referred to as the constant humidity sample) and the constant temperature immersion sample (referred to as the soaking sample) of urease in the unconfined compressive strength test is 1.0 mol/L, and the compressive strength of the soaking sample is 4.9 MPa, which is 1.56 times that of the constant humidity sample; the optimum addition ratio of the lignin-improved constant humidity sample is 3%, and its compressive strength is 2.07 Mpa; the optimum addition ratio of the samples immersed at constant temperature is 4%, and the compressive strength is 3.05 MPa; when urease combines with lignin to improve silt, 4% is the best lignin addition ratio, the compressive strength of the constant humidity sample reaches 1.57 Mpa and the compressive strength of the soaking sample reaches 3.75 MPa; in the dynamic triaxial multi-stage cyclic load test, all samples were cured at constant humidity sample, and in the urease modified silt scheme, 1.0 mol/L was the optimal cement concentration; in the scheme of improving silt with lignin, 3% is the optimal addition ratio; when 1.25 mol/L cementation solution plus urease crude extract is combined with different ratios of lignin in the experimental scheme, 3% is the best lignin addition ratio.

## 1. Introduction

In the field of geotechnical engineering, soil treatment has always been a very important research topic. Silt, sand and sand gravel are the most representative soil layers in Beijing [1]. The defects in the engineering properties of silt are that it is prone to liquefaction, shifting sand and other problems [2]. In order to make the soil meet the actual engineering needs, different degrees of improvement should be carried out on the unfavorable soil [3,4,5].

The method of consolidating soil based on inducing calcium carbonate precipitation is a soil improvement technology that has emerged in recent years, due to its advantages of simple construction [6,7], environmental friendliness [8,9], low cost [10] and easy control [11], and it has received extensive attention from the academic community. However, the MICP technology based on microbial induced calcium carbonate precipitation has problems such as biosafety risks [12], insufficient cementation of fine-grained soils [13,14] and the need for an aerobic environment for bacterial growth [15,16]. Meanwhile, micro-organisms are also able to create occluded gas bubbles in the soils that can be trapped if fines content is present. This limits the large-scale application of MICP technology in the field. Therefore, some scholars have proposed enzyme-induced calcium carbonate precipitation (EICP) technology to directly use urease to replace MICP, which also brings new research and application ideas to bio-geotechnical soils. The principle of EICP technology is to mix urease and calcium from urea into the soil [17,18,19,20]; it promotes the decomposition of urea into NH^4+^ and CO_3_^2−^, and finally forms calcium carbonate precipitation in an alkaline environment to solidify the soil. At present, the method for extracting urease is mainly from the seeds of legumes and bacteria; the supernatant of the solution, that is, the crude extract of urease, can be obtained via low-temperature high-speed centrifugation of urease in plant cells [21,22,23]. However, urease from strains not only requires low-temperature high-speed centrifugation to obtain extracellular urease [24]; it is also necessary to extract intracellular urease using disrupting methods such as ultrasound or lysozyme [25,26]. EICP technology directly extracts urease (acting as a catalyst) that catalyzes urea, thereby avoiding the drawbacks of MICP technology and improving the activity of urease [27]. The process of urease-induced carbonate precipitation is affected by many factors, including the source [28,29] and urea concentration [30,31], different calcium sources [32,33] and the environment factor [34]. Later, scholars added soil conditioners to improve soil strength in the study of EICP technology for soil improvement experiments, including skimmed milk powder [35], casein [36], polyvinyl alcohol fiber [37], bentonite [38] and nan-montmorillonite [39]; the improvement principles and effects of different additives in EICP treatment were discussed. The performance improvement effect of soil samples after EICP treatment is mainly measured using mechanical tests such as the unconfined test [40], the triaxial test [41,42] and the direct shear test [43]. The current EICP technology has been applied in engineering applications including windbreak and sand fixation [44,45], seepage prevention and leakage stoppage [46,47,48], foundation improvement [49,50,51,52,53,54,55], soil slope reinforcement [56,57], crack repair [58,59] and cultural relic restoration [60], and other fields have achieved good results and have better prospects. However, the direct use of urease to catalyze the hydrolysis of urea and then the formation of calcium carbonate lack nucleation sites that are conducive to crystal formation; in order to make up for this defect in EICP technology, this paper introduces lignin, a natural, efficient and environmentally friendly soil conditioner, to act as a nucleation site to improve soil.

This paper mainly studies the improvement effect of urease, lignin and their mixture on silt. Through the results and phenomena of unconfined compressive strength and the dynamic triaxial test, the improvement effect of different improvement methods and maintenance methods is analyzed, so as to obtain the optimal value of the cement or lignin when EICP technology, lignin alone or EICP and lignin are used to improve silt.

## 2. Materials and Methods

Enzyme-induced calcium carbonate precipitation (EICP) technology is a process similar to MICP mineralization that directly extracts urease, which catalyzes urea, as a catalyst. Urease is an enzyme that specifically catalyzes the hydrolysis of urea and is widely found in microorganisms and animal and plant tissues; different types of urease have different catalytic effects. The cultured strain in this paper is *Sporosarcina pasteurii*, and the cultured *Sporosarcina pasteurii* is subjected to cell lysis to obtain intracellular urease; the extracellular urease of *Sporosarcina pasteurii* can often be obtained from the supernatant after low-temperature high-speed centrifugation. The acquisition of intracellular urease can be carried out using the ultrasonic cell lysis method, and *Sporosarcina pasteurii* may also be disrupted after treatment with lysozyme.

### 2.1. Experimental Materials

The strain used in the test was the second-generation *Sporosarcina pasteurii* strain, numbered ATCC 11859, the optimum pH was around 7.3 and the optimum temperature was 30 °C. It belongs to aerobic Gram-positive bacteria, and the culture time is 1–2 d. The nutrient used to cultivate *Sporosarcina pasteurii* was Nutrient Broth basal medium, the ingredients were peptone 10 g/L, beef extract powder 3 g/L and sodium chloride 5 g/L and the pH was 7.2 ± 0.2.

During the proliferation of *Sporosarcina pasteurii*, one of the metabolites is an enzyme called urease. Urease is a highly specific enzyme that catalyzes the hydrolysis of urea into ammonia and carbon dioxide, and its optimum pH is around 7.4.

(1) $$\mathrm{CO}{\left({\mathrm{NH}}_{2}\right)}_{2}+H_{2}O\to {\mathrm{CO}}_{2}+2{\mathrm{NH}}_{3}$$

Some studies have shown that the conductivity change value of 1 ms/min corresponds to the urea hydrolysis amount of 11 mM, and the relational formula is as Formula (2).

(2) U=SKA

In the formula, *S* is the dilution ratio of the bacterial solution; *K* is the conductivity change (ms/min); *A* = 11.11 and *U* is the urease activity value (mM.ms/min).

**Figure 1:**
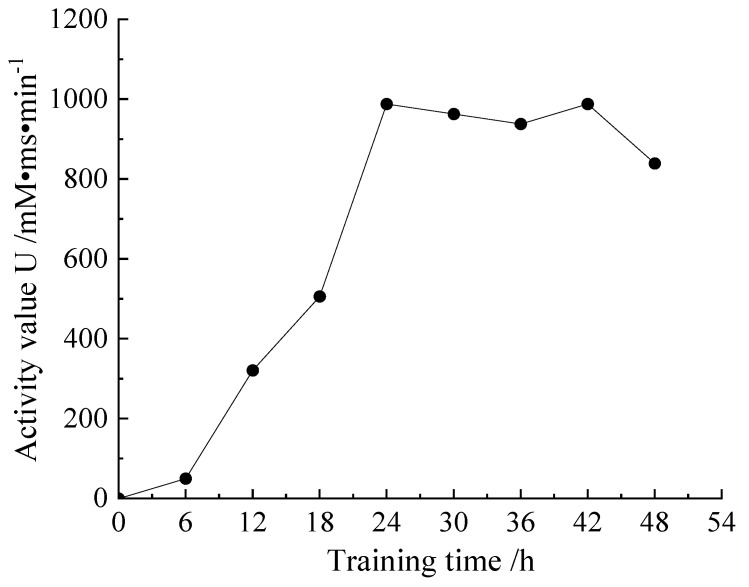
Urease activity curve.

Figure 1 is the urease activity curve, the dilution factor of the bacterial solution is 10 and the measured conductivity change value k is the average value 5 min after adding the bacterial solution. Urease activity peaked at 24 h: 988 mM.ms/min.

The soil used in the experiment was sandy silt, which was taken from a construction site in Beijing. Its standard penetration number N was 5–9, and the density was slightly dense; after natural air-drying on site, it was khaki. The test soil was naturally dried in the room before being used for sample preparation, and then the basic physical properties of the obtained test soil were studied, (its physical properties are shown in Table 1), and the particle size gradation curve is shown in Figure S1.

### 2.2. Sample Preparation and Maintenance

The soil used for sample preparation needed to be pre-mixed with soil, urease, lignin and cementation solution and other substances before sample preparation; therefore, the sample preparation method is called the mixing method. In this article, there were mainly two sizes of sample maker, 39.1 mm × 80 mm and 50 mm × 100 mm, respectively; the former was used for the unconfined compression test, and the latter was used for the dynamic triaxial test. The number of substances consumed to prepare the samples is shown in Table 2.

There were two main ways of maintaining the sample: constant temperature and humidity curing and constant temperature soaking curing (corresponding samples are referred to as the constant humidity sample and the soaking sample). For the samples cured at constant temperature and humidity, the soil used was dried and sieved indoors and the solution was as follows: solution (cementation liquid and bacterial liquid) = 1000 g: 200 mL ratio. Before sample preparation, the soil and bacterial liquid should be mixed evenly. Then, the cementation liquid was added and mixed to prepare the samples; after the samples were prepared, they were wrapped with plastic wrap to prevent the moisture of the samples from evaporating and to keep the moisture content of the samples consistent. The curing temperature of the sample was 30 °C. The mass of each sample after curing was 382 ± 5 g (the sample weight range for dynamic triaxial), and the density change was within 1.31%.

The production process of the constant humidity sample and the immersion sample was consistent, and the constant temperature immersion method was used to prepare the samples first and then put them into the corresponding cementation solution for curing. However, the curing method of constant temperature immersion is difficult to ensure the moisture content of the sample, so the mechanical properties are generally tested directly after drying. In the dynamic triaxial test, constant humidity samples do not need to be dried at low temperature; keep the well-maintained state and directly carry out the dynamic triaxial test and all of them will be carried out under the same conditions. They do not need to be consolidated or drained. The confining pressure was 50 kPa.

### 2.3. Unconfined Compressive Strength Test Equipment

In the unconfined compression test, the compressive strength and failure form of the two samples made by mixing with constant temperature and humidity curing and mixing with constant temperature immersion, curing after drying, were mainly studied, and both samples were dried at a low temperature of 60 °C to obtain samples.

The universal testing machine used in this test was the electronic universal material testing machine RGM-100KN (Figure S2); the testing machine was controlled by a computer throughout the process and it could store and analyze data, print analysis reports and conduct multiple tests. Its measurement system had three parts: load measurement, specimen deformation measurement and moving beam displacement measurement. The sample was damaged under pressure under the action of axial pressure, and the displacement control method was adopted at 0.05 mm/min. In the test, the lower pressure plate (indenter) position was mainly used to test the sample. The unconfined force compressive test was carried out on the sample using this testing machine to determine the compressive strength q_u_ of the sample.

### 2.4. Dynamic Triaxial Test Equipment

In order to study the mechanical characteristics and performance differences in the improved silt under dynamic loads, dynamic triaxial tests were carried out on the improved samples. In the dynamic triaxial test, the samples under constant temperature and humidity curing did not need to be dried at low temperature, and the dynamic triaxial test was directly carried out in a well-cured state; they were all carried out under the same conditions: no consolidation and no drainage, and the confining pressure of the shear test was 50 kPa.

The dynamic triaxial test instrument used in this test was the KTL dynamic triaxial system (Figure S3); the system was mainly composed of five parts, as shown in Figure S3: axial loading equipment with pressure chamber and counterweight mechanism, confining pressure controller, back pressure controller and 8-channel high-speed control and acquisition equipment. The applicable sample size was 50 mm × 100 mm, and the system applied the load through the movement of the base on which the sample was loaded. In this dynamic triaxial test, under the condition of a confining pressure of 50 kPa, the axial load was applied in stages. For the sine-type wave, the frequency was 1 Hz, the amplitude was 60% of the central load, and the vibration frequency of each stage was 10; until the specimen failed or the strain reached 6%, the loads applied step by step were 50 N, 100 N, 200 N, 300 N, 400 N and 500 N, respectively.

## 3. Experimental Schemes for Improving Silt with Urease–Lignin

This paper directly studies the effect of urease on silt mineralization, at the same time that lignin is introduced, in order to make up for the defect that EICP technology does not have nucleation sites during the crystallization of calcium carbonate. In order to quantitatively analyze the differences in the properties of silt before and after improvement and under different improvement conditions, the silt was made into corresponding sizes and subjected to mineralization; then, unconfined and dynamic triaxial tests were used to study the improvement effect. In this paper, the silt before and after improvement is analyzed mainly through two different maintenance methods.

### 3.1. EICP Optimal Concentration Test Scheme

The urease studied in this paper is obtained by culturing *Sporosarcina pasteurii* and then breaking the cell; urease was obtained by blasting *Sporosarcina pasteurii* with lysozyme with a culture time of 24–32 h in Section 2.1. The lysozyme used in this paper is egg white lysozyme, a white powder that is soluble in water and has a good hydrolysis effect on Gram-positive bacteria. Dissolve the lysozyme in pure water, using the dosage 1 g:1000 mL bacterial liquid; then, act on the bacterial solution and obtain the crude extract of urease after 1 h in a constant temperature water bath. The obtained crude urease extract should be used for sample preparation immediately. The moisture content of the prepared sample should be 17–25%, between the plastic limit and the liquid limit; Table 3 shows the preparation concentration parameters of the cementation solution.

### 3.2. Test Scheme of Optimal Addition Ratio of Lignin

The lignin used in this paper was purchased from Hefei BASF Biotechnology Co., Ltd. After dissolving in water, it is easy to attract Ca^2+^, making it crystallize and aggregate on the surface. First, the unconfined compressive test was used to determine the appropriate range of the addition ratio of the improved silt, setting the experimental groups with addition ratios of 0, 3%, 6%, 9%, 12% and 15%. Using the unconfined compressive strength test, the maximum axial load F_max_ range that the samples with different lignin addition ratios could bear was determined, as shown in Table 4.

Thus, the optimum range of 0–6%, suitable for this test silt, was preliminarily determined. In order to precisely reduce the optimal addition ratio of lignin, in the following experiments, lignin groups with different addition ratios were set; they were 1.5%, 3%, 4%, 5% and 6%. Among them, the soil consumption of each of the three samples for the unconfined compressive strength test was about 480 g. For the samples used in the dynamic triaxial test, the soil consumption for each of the three samples was about 960 g.

## 4. Results and Analysis

The improvement effect is mainly verified via mechanical testing. There are mainly two kinds of mechanical tests used in this paper: the unconfined compressive test mainly tests the compressive strength of the sample and observes the damage of the sample; the dynamic triaxial test is used to compare and analyze the differences in the mechanical properties of the samples under dynamic loads under different schemes. The analysis is carried out from four aspects: the failure mode of the sample, the accumulated strain, the elastic modulus and the maximum load that the sample can bear.

### 4.1. Unconfined Compression Test

#### 4.1.1. EICP(E) Scheme Effect Analysis

The mixing temperature was kept constant and the humidity-curing urease was combined with different concentrations of cement samples. After, the silt was improved with urease obtained from *Sporosarcina pasteurii*; the improved effect is shown in Figure S4a. From left to right, the concentration of the cementation solution is 0.5 mol/L, 0.75 mol/L, 1.0 mol/L, 1.25 mol/L and 1.5 mol/L. The failure shapes of the samples are mainly different crack penetration failures, Z-shaped and I-shaped samples remain relatively intact after failure, and the crack development of the samples is relatively consistent, with less local drop.

Samples of the constant temperature immersion method and the specimens under this test scheme were obtained using the immersion method; under the action of the axial pressure without lateral restraint, the failure mode of the sample is mainly the loss of the overall compressive strength after the crack penetrates, and the individual samples have local drop damage, as shown in Figure S4b. Among them, the samples passing through a 1.25 mol/L cementation solution showed a more typical failure shape.

**Figure 2:**
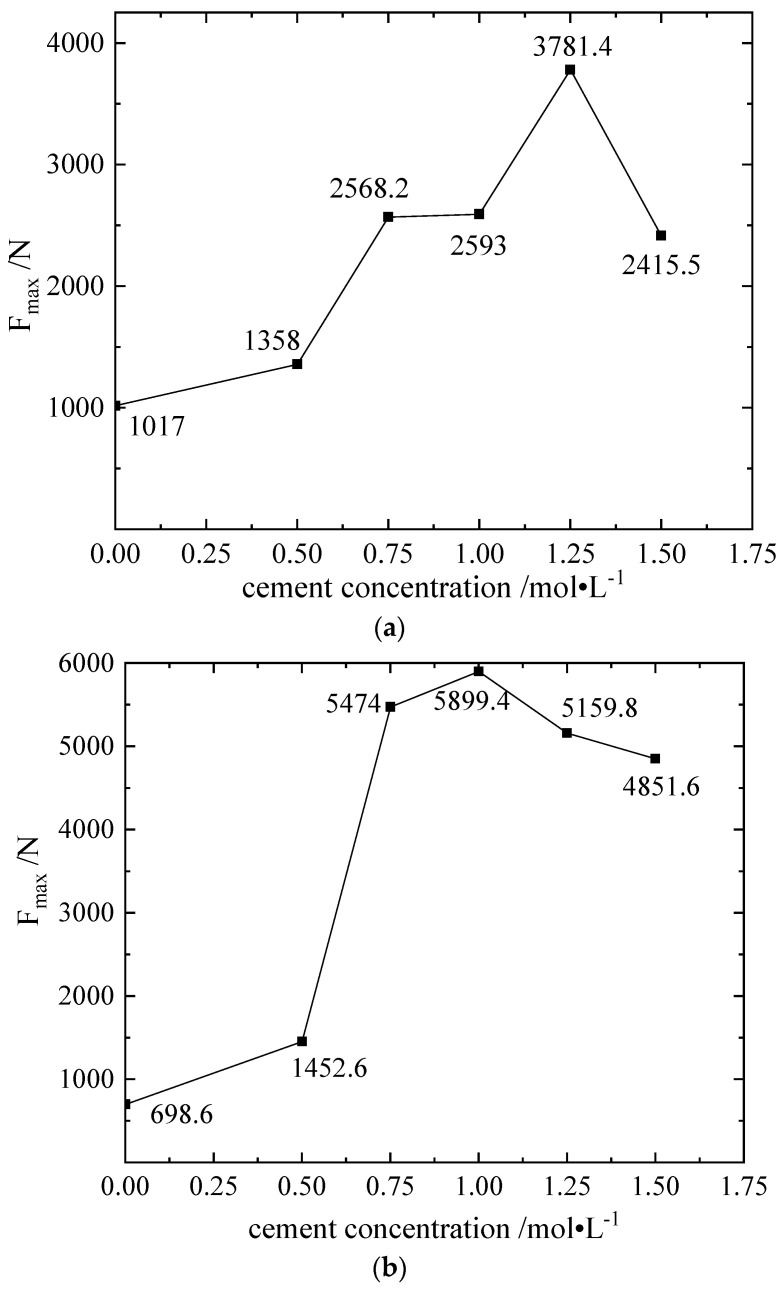
Concentration of cementitious liquid and maximum axial force Fmax of different samples in scheme E. (**a**) Concentration and F_max_ diagram of cementitious solution of constant humidity sample in Scheme E. (**b**) Figure of the concentration and maximum axial force F_max_ of the soaking sample in Scheme E.

From Figure 2b, compared with the blank group, for any cement concentration between 0.5–1.5 mol/L, the compressive properties of the improved silt were improved. The improvement effect reaches the maximum value when the cementation solution concentration is 1.25 mol/L, the axial pressure is 3781.4 N, the compressive strength q_u_ is 3.15 MPa and it is 3.7 times that of the blank group and 1.8 times that of the corresponding *Sporosarcina pasteurii* mixed with 1.28 mol/L cementation solution.

#### 4.1.2. Analysis of Improvement Effect of Lignin (L) Scheme

The samples were prepared via the mixing constant temperature and humidity method, and the samples modified by lignin showed three failure modes after compression failure, as shown in Figure S5a. The sample showed the phenomenon of bulging damage, that is, cracks appeared at a certain height along the side of the sample; this phenomenon appeared in some samples with 4%, 5% and 6% lignin additions, but not in the lower additions of 1.5% and 3%. While the specimen is compressed longitudinally, the transverse expansion occurs to generate tensile stress, indicating that the specimen has a certain compressive strength, but that the tensile strength is small. When the proportion of added lignin was high, the sample appeared to show tensile stress failure (drum waist failure), indicating that in the samples of the mixing constant temperature and humidity curing method, a too-high lignin addition was not conducive to the bonding of the samples.

**Figure 3:**
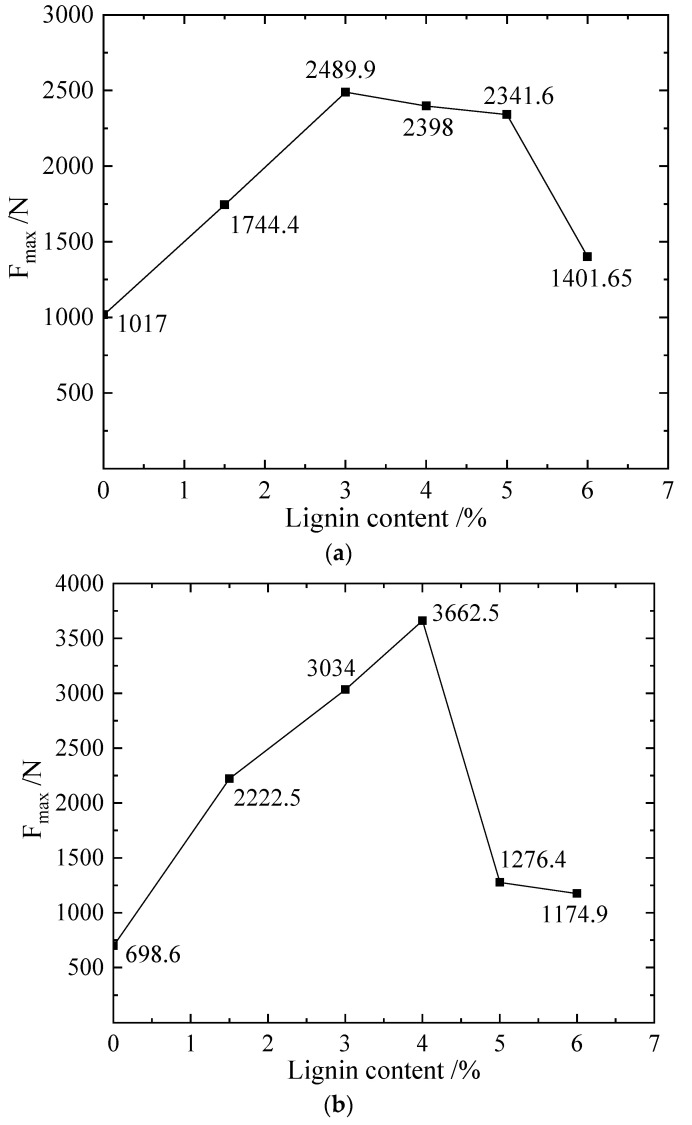
Maximum bearing capacity F_max_ and lignin content of different samples in L scheme. (**a**) Diagram of maximum bearing capacity F_max_ and lignin content of constant humidity sample in Scheme L. (**b**) F_max_ and lignin addition ratio of solution L soaking sample.

Figure 3a shows the change curve of the maximum axial force under different lignin content, from the perspective of the improved mechanical properties of the sample; the addition of lignin can improve the compressive performance of the sample. However, the samples obtained by direct mixing and curing will not improve the cohesion of the samples; on the contrary, it will decrease. From the perspective of the maximum axial pressure that the sample can bear, the maximum compressive strength reaches the maximum value of 2.07 MPa; when the ratio of lignin is 3%, the axial pressure is 2488.75 N. When the addition of lignin is between 0–6%, the increase is large before reaching 3%, and the decrease is slower when it is between 3–5%. The above can show that for the silt studied in this experiment, the addition ratio of lignin has a better improvement effect when the ratio of lignin is 3%, and its compressive strength is 2.5 times that of the original.

When the unconfined compressive strength test of the sample obtained using the constant temperature immersion method is carried out, it is mainly manifested as local failure, crack development and penetration, as shown in Figure S5b. There are also two main types of crack development: a Z-shaped crack formed with the end face, and an I-shaped crack formed with the end face.

The lignin sample obtained using the constant temperature soaking method has a compressive strength of 3.05 MPa at 4%, and its maximum axial force can reach 3662.5 N, as shown in Figure 3b. When using the constant temperature soaking method for curing, lignin is soluble in water due to the contact between the sample and water; theoretically, the content of lignin is 4%, but the actual lignin content in the sample is less than 4%. The test soil is fine-grained soil, and it is also a static water flow under the constant temperature immersion curing method; therefore, if lignin is applied to the fine-grained soil, attention should be paid to the loss of lignin.

#### 4.1.3. Analysis on the Improvement Effect of EICP—Lignin (E-L) Scheme

As shown in Figure S6a, the sample was obtained through constant temperature and humidity curing; in the unconfined compressive test, by adding a proportion of 1.5% to 6% of the sample the degree of fragmentation increases and it can be seen that after the test of the 5% group is damaged by compression, it is difficult to maintain the whole, and the degree of fragmentation is serious.

**Figure 4:**
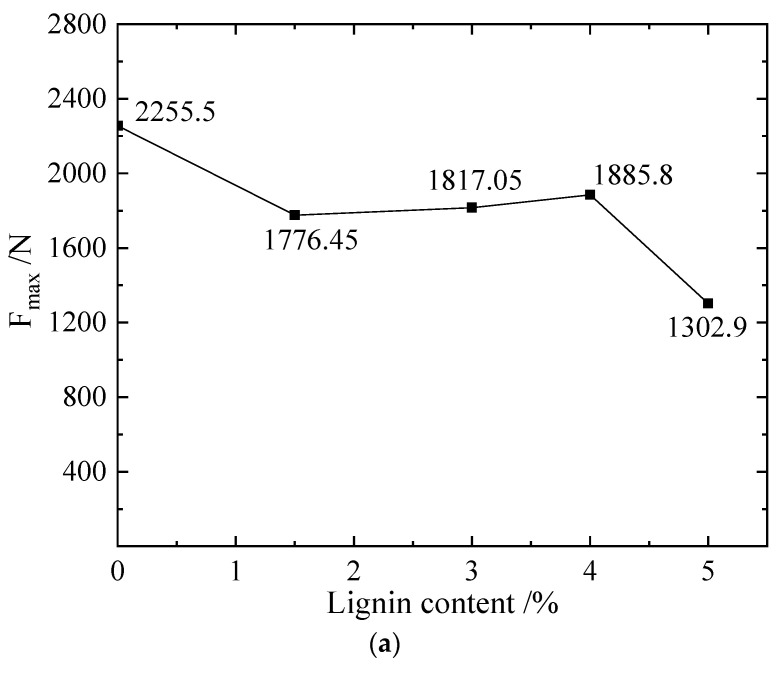

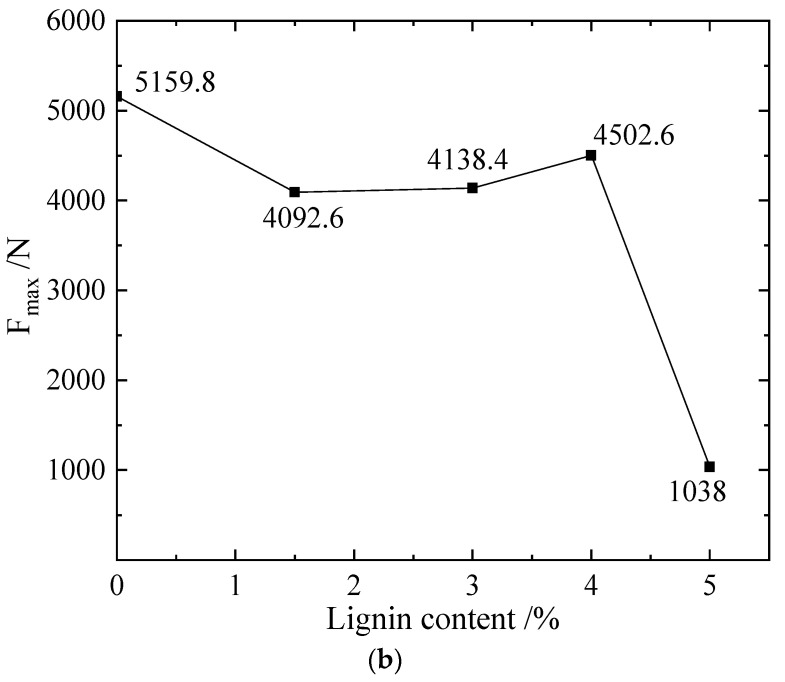
The ratio of lignin addition and the maximum axial force F_max_ for different samples of E-L scheme. (**a**) Diagram of lignin addition ratio and maximum axial force F_max_ of constant humidity sample. (**b**) Diagram of lignin addition ratio and maximum axial force F_max_ of soaking sample in E-L scheme.

As shown in Figure 4a, the blank group was 1.25 mol/L cementation solution + urease, and the improvement effect of each group under this scheme on silt was smaller than that of the blank group; this shows that the addition of lignin did not improve the cementation effect of this group, but hindered the cementation effect of urease. The maximum compressive strength obtained from this hybrid test was 1.57 MPa. The improvement strength of the subsequent silt showed a trend of increasing and then decreasing with an increase in lignin; this shows that the addition of a certain amount of lignin is indeed beneficial to the improvement of the compressive strength of the sample, while the addition of an excessively high proportion has the opposite effect.

The samples of this group obtained using the constant temperature immersion curing method, as shown in Figure S6b, are relatively complete two parts after compression failure, without too many loose particles; this is a typical compressive shear failure with a typical z-shaped crack. In the sample shown in Figure S6b, the upper failure surface develops from the left, and the lower failure surface develops from the right, which is also a z-shaped crack. There was no form of bulging failure in the test, and no parallel cracks developed through the two end faces of the sample, indicating that the cementation effect of this group is good.

As shown in Figure 4b, compared with the blank control group of 1.25 mol/L cement-urease, the compressive strengths of the samples added with different concentrations of lignin were lower than those of the blank control group; this indicates that the addition of lignin hindered the urease cementation in this cementation concentration group. The decrease in compressive strength caused by the hindering effect cannot be compensated by the increase in lignin content; before the lignin reached the optimal value, the compressive strength of the sample was still lower than that of the blank group, and the compressive strength was 3.75 MPa, and in the group with 5% lignin content, the compressive strength value decreased significantly. This shows that in the samples using the constant temperature immersion curing method, the addition ratio of lignin 5% is a critical value, and 5% will cause a significant decrease in the compressive performance of the samples.

### 4.2. Dynamic Load Performance Test Considering Confining Pressure

#### 4.2.1. Analysis of Improvement Effect of EICP(E) Scheme

The concentrations of the cementation solution from left to right in Figure S7 are 0.5 mol/L, 0.75 mol/L, 1.0 mol/L, 1.25 mol/L and 1.5 mol/L. In this group, slant cracks (a–c) and X-shaped cracks (d,e) appeared in sequence when the specimens were damaged by dynamic loading.

**Figure 5:**
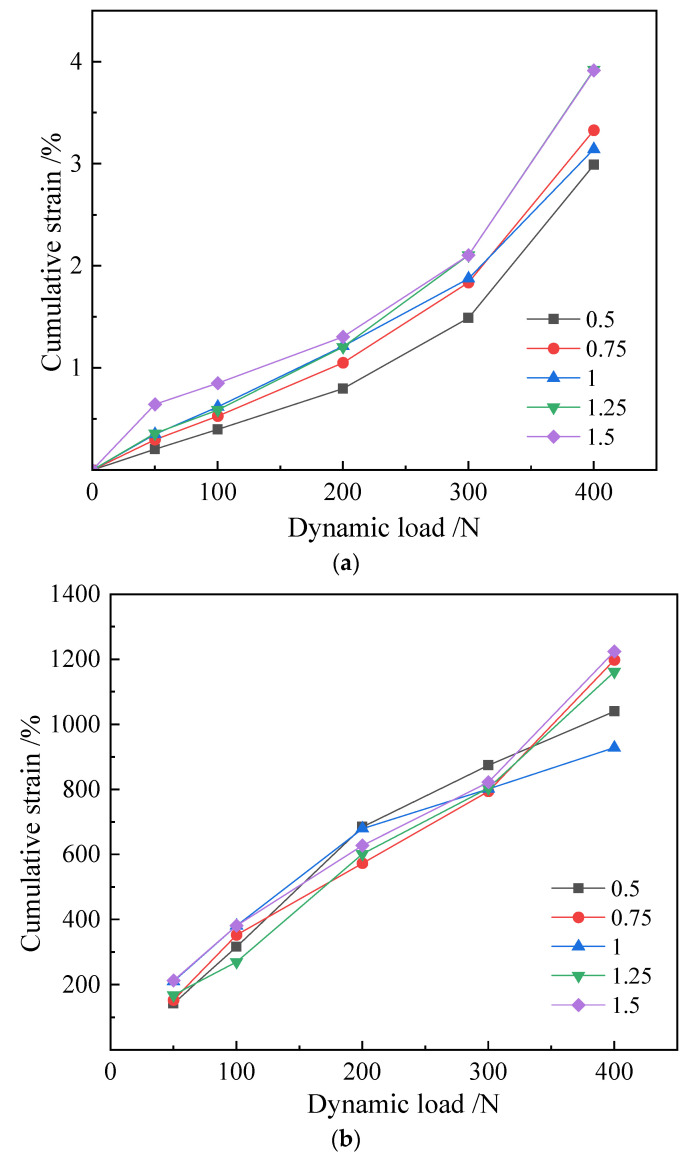
E scheme sample. (**a**) Cumulative strain curve; (**b**) resilience modulus Ed versus dynamic load curve.

According to the analysis of the cumulative strain dynamic load curve of this group in Figure 5, it can be preliminarily judged that the improvement effect is ranked from high to low as follows: 0.5 > 0.75 > 1.0 > 1.25 > 1.5; the overall performance is that the low concentration group is better than the high concentration group. The trends of the cumulative strain dynamic load curves of each group are basically the same, and the overall low concentration is higher than the dry concentration of the cementitious liquid group. The elastic modulus is basically the same as that of the applied axial loads at all levels; the elastic modulus of the gap with the dynamic load increasing step by step decreases gradually. At the same time, when the dynamic loads at all levels are cyclically applied, the elastic modulus of the specimen also increases.

**Figure 6:**
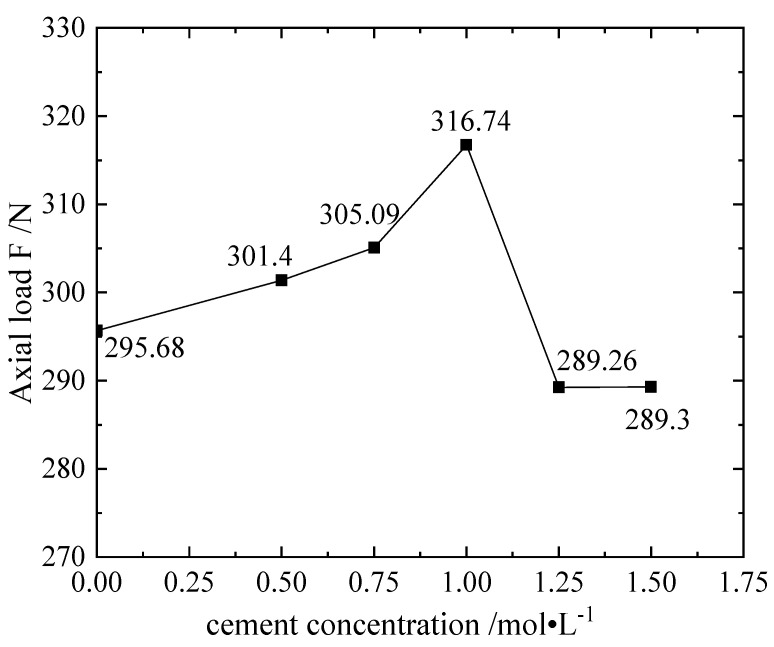
Sample of E scheme: F diagram of cementation solution concentration and axial load.

As shown in Figure 6, the 1.0 mol/L group has the maximum bearing axial load value of 316.74 N, which is combined by urease with different cementation solution concentrations; overall, the load-carrying capacity of the samples modified by the cement is not much different from that of the unmodified samples. However, considering the cumulative strain curve, the elastic modulus and the maximum axial load that can be tolerated, the optimal concentration is in the group of 1.0 mol/L and below. The trend of combining *Sporosarcina pasteurii* with different concentrations of cement is basically the same, and the overall performance of the two is as follows: the optimal concentrations obtained under dynamic triaxial multi-stage cyclic loading are all less than 1.25 mol/L obtained in the unconfined compressive strength test. The difference between the two test samples is that the moisture content of the sample used in the dynamic triaxial test is about 20%, and the sample used in the unconfined test is a low-temperature drying sample.

#### 4.2.2. Analysis of Improvement Effect of Lignin (L) Scheme

Figure S8 shows the samples with the addition ratios of the blank group, 1.5%, 3%, 4%, 5%, and 6%, in order. It is obvious that when the lignin addition ratio is low, slant cracks appear when the sample is damaged; when the addition ratio exceeds 3%, the sample does not have cracks under multi-level loading, but only has drum-shaped deformation formed by compression, which has a larger plastic deformation capacity than the blank group.

**Figure 7:**
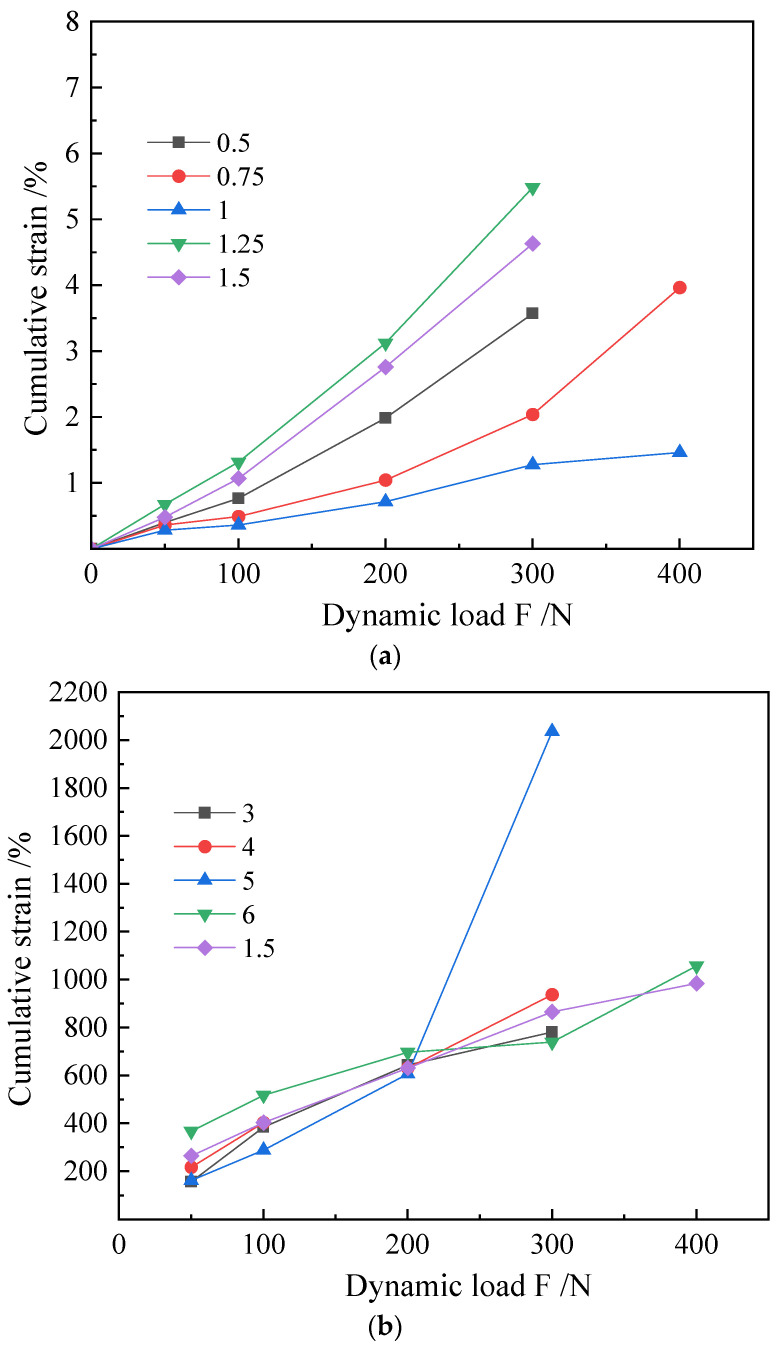
L scheme sample. (**a**) Cumulative strain curve, (**b**) resilience modulus Ed and dynamic load curve.

According to the analysis of the cumulative strain dynamic load curve, Figure 7 was obtained by statistics and the 3% group is the optimal curve. The proportion of lignin added and the cumulative strain of the sample show an inverse V trend: the deformation resistance of the samples increased with an increase in the lignin addition ratio, and after reaching 3%, the deformation resistance capacity decreased with an increase in the lignin addition ratio. Among them, the sample with a lignin addition ratio of 6% had a large amount of longitudinal compression due to the large axial pressure applied; however, observing the sample after compression deformation shows that the sample has no damaged surface, indicating that under the action of axial compression and confining pressure, the sample is compacted and has a higher elastic modulus.

It can be seen that when lignin is used to improve this type of silt in practical engineering, 3% is the optimal addition ratio when a single addition of lignin is used for improvement. If a certain settling is allowed, it can be compacted by adding lignin and supplemented by mechanical methods; at this time, 6% is a good addition ratio.

**Figure 8:**
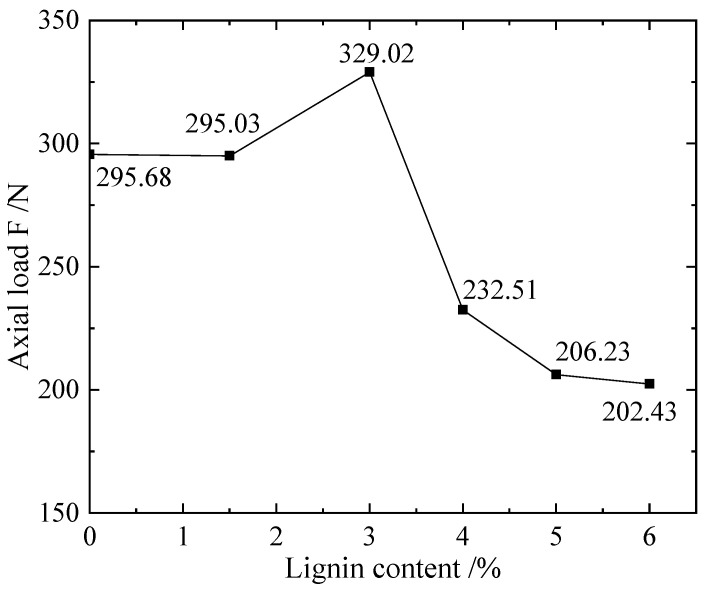
Sample of L scheme: lignin addition ratio and axial load F diagram.

From Figure 8 of the maximum axial load, it can be seen that the strength of the silt in this test can be greatly improved when the lignin addition ratio is 3%; however, when the addition ratio is too high, its bearing capacity is worse than that of the unimproved silt. After comprehensive analysis of cumulative strain dynamic load, elastic modulus and the maximum dynamic load that the sample can bear, 3% is found to be the optimal lignin addition ratio for the experimental silt. 

#### 4.2.3. Analysis on the Improvement Effect of EICP—Lignin (E-L) Scheme

From left to right in Figure S9, the addition ratios of lignin are 1.5%, 3%, 4% and 5%. In this group, when the proportion of lignin added was 1.5% and 3%, slant cracks appeared when the samples were damaged, while when the groups with the addition ratio of 4% and 5% were subjected to dynamic loads, only drum-like deformation appeared. It is basically the same as the failure form when lignin is used alone to improve silt; the lignin content above 3% is mainly due to drum deformation, and the group below or equal to 3% is mainly shear failure.

**Figure 9:**
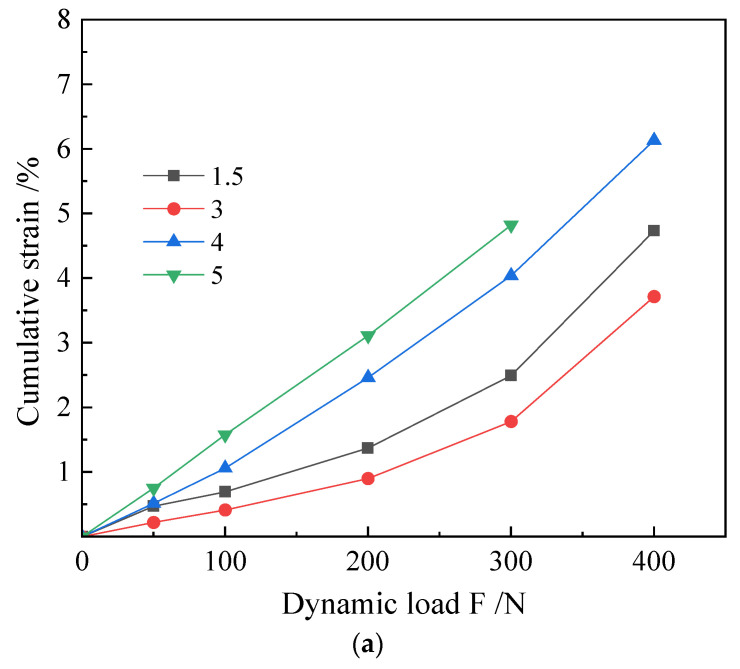

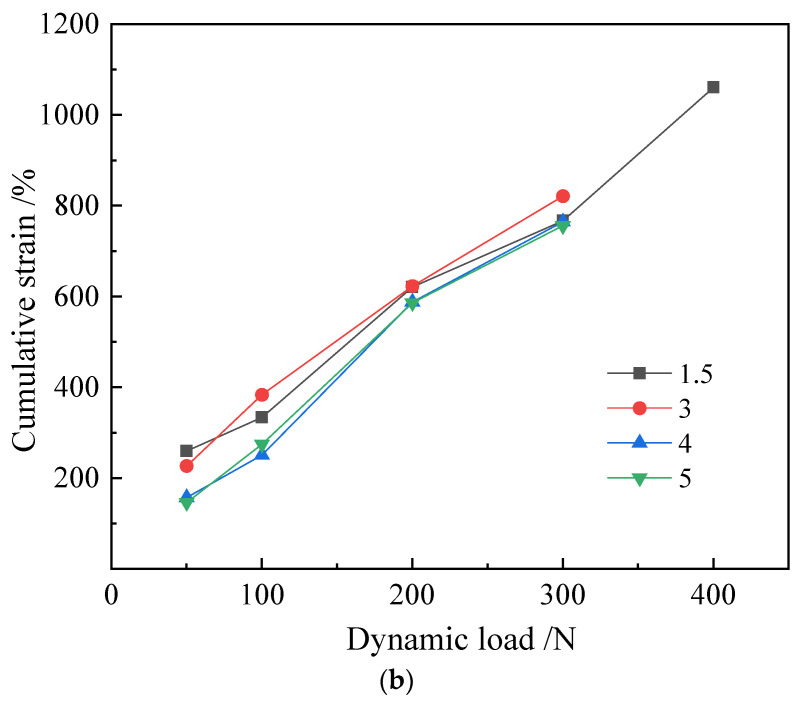
E-L scheme sample. (**a**) Cumulative strain curve, (**b**) resilience modulus Ed and dynamic load curve.

As shown by the cumulative strain curves of this group in Figure 9, the optimal lignin addition ratio was 3%, followed by 1.5%, 4% and 5%. On the whole, it still shows that the improvement effect of silt with a low addition ratio is better than that of the high addition ratio group. The curve corresponding to the addition ratio of 5% has a good linear correspondence between the cumulative strain and the dynamic load. From the statistical analysis of the elastic modulus, 3% is the optimal curve, followed by 1.5%, which has a good correspondence with the cumulative strain curve.

**Figure 10:**
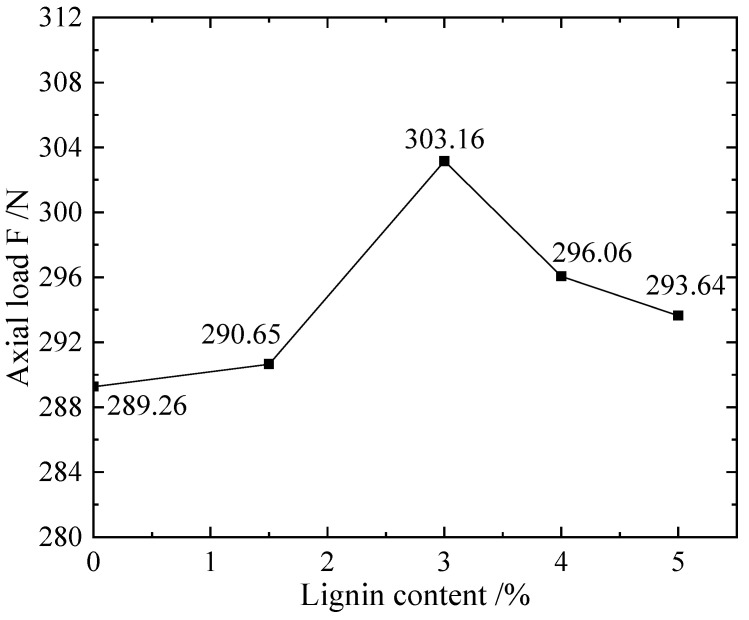
E-L scheme sample: the ratio of lignin added and the axial load F diagram.

As shown in Figure 10, the silt soils with different lignin concentrations were combined by EICP; it can be known from its maximum axial load under multi-level cyclic loading that when the lignin content is 3%, there is a higher loading value of 303.16 N. From the comprehensive cumulative strain curve, the elastic modulus and the maximum axial load curve that the sample can bear, it can be seen that 3% is the optimal value when EICP is combined with lignin to improve silt.

## 5. Conclusions

This paper takes the silt in Beijing area as the improvement object, and sets up plans such as EICP technology, lignin improvement alone or mixed improvement silt, etc.; the unconfined compressive strength and dynamic triaxial test were used to evaluate the performance of the improved soil.

i. Through the unconfined compressive strength test of the samples obtained by the constant temperature and humidity curing method and the constant temperature soaking method, the influence of the concentration of cementation solution and lignin content on the urease-modified silt was analyzed; the results show that the optimum cementation solution concentration of the urease-modified silt sample soaked at a constant temperature is 1.0 mol/L, and the compressive strength is 4.9 MPa, which is 1.56 times that of the constant humidity sample. The lignin-modified silt samples were soaked at constant temperature, the optimal addition ratio of lignin was 4%, the compressive strength was 3.05 MPa, the optimum addition ratio of lignin for the samples under constant temperature and humidity curing was 3% and the compressive strength was 2.07 MPa. In the scheme of urease combined with lignin to improve silt, under the cementation solution concentration of 1.25 mol/L, 4% was the optimal lignin addition ratio, and the compressive strength of the constant temperature and humidity samples reached 1.57 MPa. The compressive strength of the samples soaked at constant temperature reached 3.75 MPa.
ii. By performing a dynamic triaxial multi-stage cyclic load test on the constant temperature and humidity curing sample, the test plan was optimized from the cumulative strain, elastic modulus Ed and maximum dynamic load of the sample. In the scheme of improving silt with lignin in different addition ratios, 3% was the optimal addition ratio; when urease and different cementation solution concentrations were used to improve the silt scheme, 1.0 mol/L was the optimal cementation solution concentration; and when 1.25 mol/L cementation solution plus urease crude extract combined with different ratios of lignin in the experimental scheme, 3% was the best lignin addition ratio.
iii. Compared with the blank control group with 1.25 mol/L cement–urease, the compressive strength of the samples added with different concentrations of lignin was lower than that of the blank group, indicating that the addition of lignin hinders the urease cementation under this cementation concentration group. The decrease in compressive strength caused by the blocking effect cannot be compensated by the increase in lignin content. Before the lignin reached the optimal value, the compressive strength of the sample was still lower than that of the blank group, with a compressive strength of 3.75 MPa. In the group with 5% lignin content, there was a significant decrease in the compressive strength value. It can be seen that the lignin addition ratio of 5% is a critical value.

## Figures and Tables

**Table 1:** Basic physical parameters of silt.

Soil Sample	Air-Dry Moisture Content W/%	Liquid Limit W_L_/%	Plastic Limit W_P_/%	Plasticity Index I_P_	Grain-Specific Gravity G_S_	Dry Density ρ_d_	Density ρ
Silt	0.682%	25.30	16.71	8.98	2.63	1.577	1.921

**Table 2:** The amount of each substance consumed in the preparation of a single sample.

	Bacterial Liquid	Soil	Cementation Solution
Unconfined	48 mL	160 g	48 mL
Dynamic triaxial	96 mL	320 g	48 mL

**Table 3:** Cementation solution configuration parameters.

Concentration mol/L	0	0.5	0.75	1.0	1.25	1.5
Anhydrous calcium chloride/g	0	55.49	83.24	110.98	138.73	166.47
Urea/g	0	30.03	45.05	60.06	75.08	90.09

**Table 4:** Relationship between lignin addition ratio and F_max._

The Proportion of Lignin Added/%	0	3	6	9	12	15
Fmax/N	1017	2489.9	1401.65	1173.28	925.9	829.8

## Data Availability

All data that support the findings of this study are available from the corresponding author upon reasonable request.

## Supplementary Materials

The following supporting information can be downloaded at: https://www.mdpi.com/article/10.3390/ma16030999/s1.
