# Supplementary material for: Experimental Study on Different Improvement Schemes of EICP-Lignin Solidified Silt
Source: Materials (Basel). 2023 Jan 21;16(3):999. doi: 10.3390/ma16030999 (PMC9919099; doi:10.3390/ma16030999)
Supplement: Supplementary file 1 [file materials-16-00999-s001.zip › materials-2156935-supplementary.pdf]

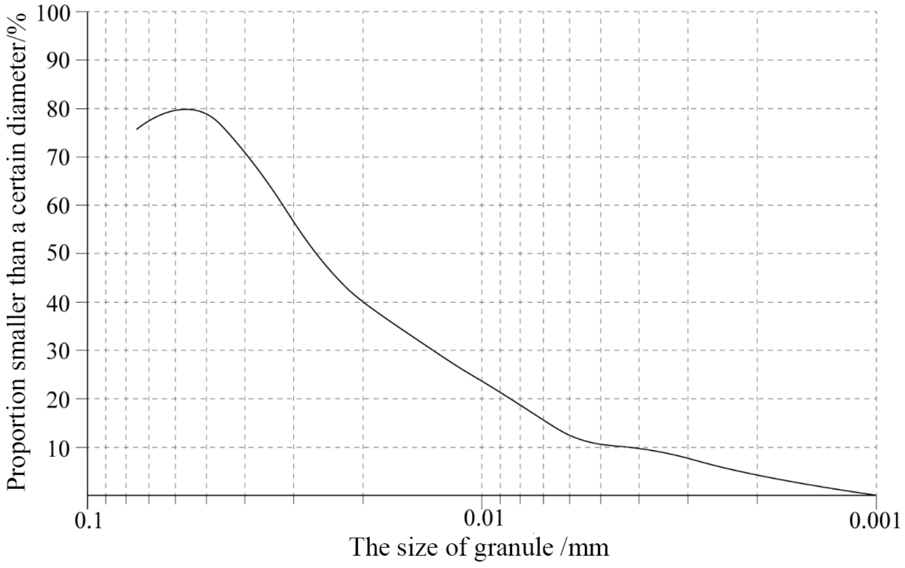

Figure S1. Particle size distribution curve.

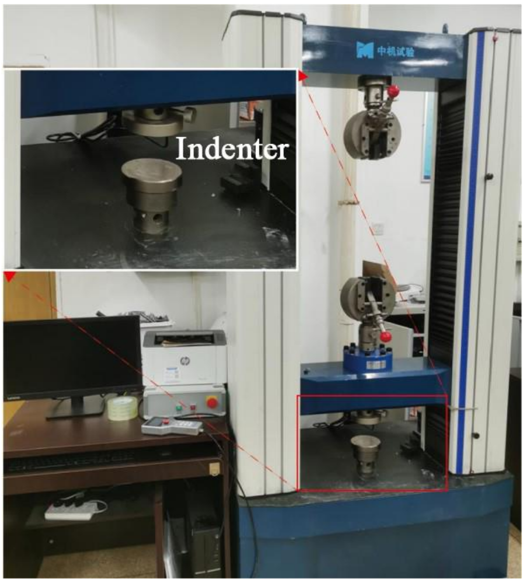

Figure S2. Electronic universal material testing machine RGM-100kN.

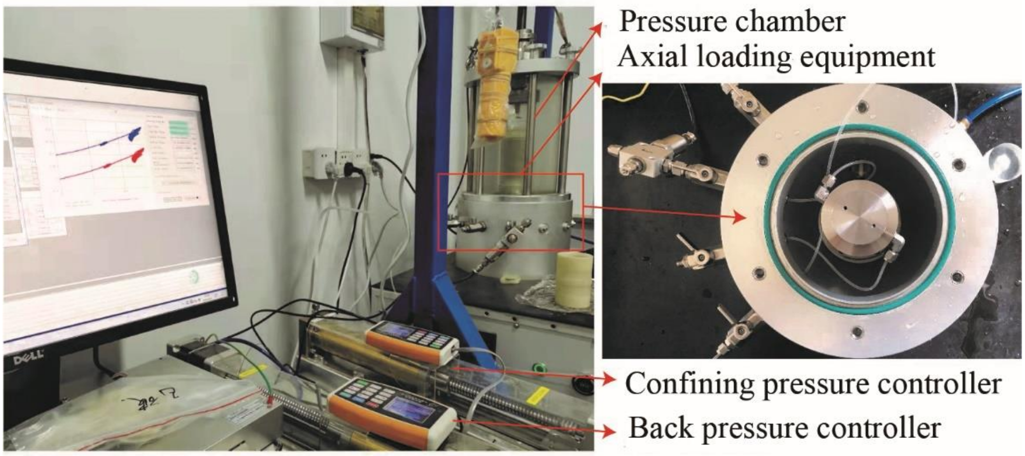

Figure S3. KTL dynamic three-axis system.

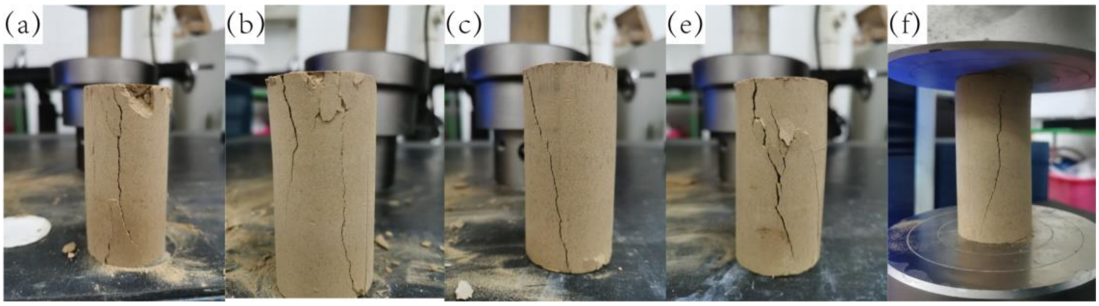

(a) The pressure failure diagram of the constant temperature sample of the scheme E

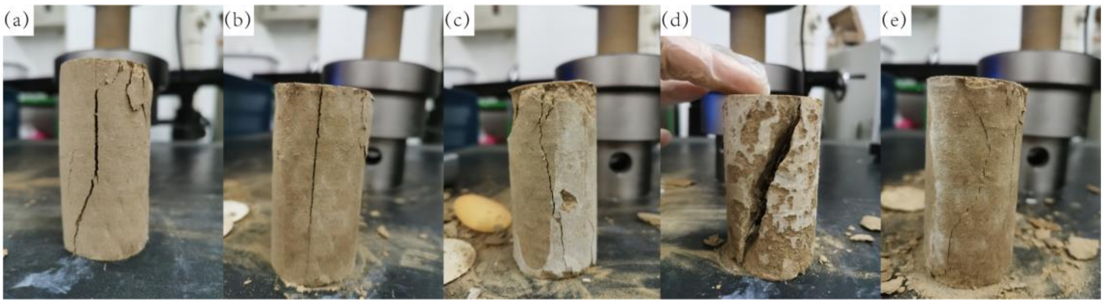

(b) Compression failure diagram of immersed samples in scheme E

Figure S4. Compression failure diagram of different samples in scheme E.

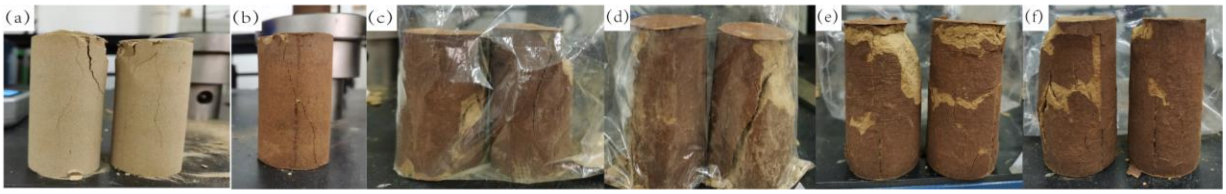

(a) Pressure failure diagram of constant temperature sample of L scheme

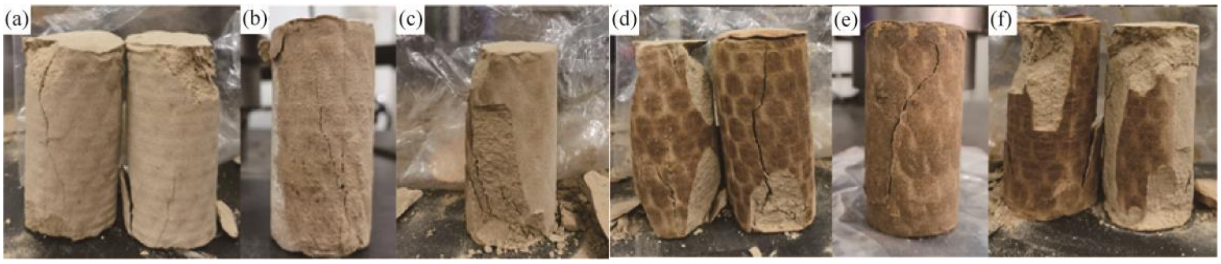

(a) Compression failure diagram of immersed samples in L scheme

Figure S5. Compression failure diagram of different samples in L scheme.

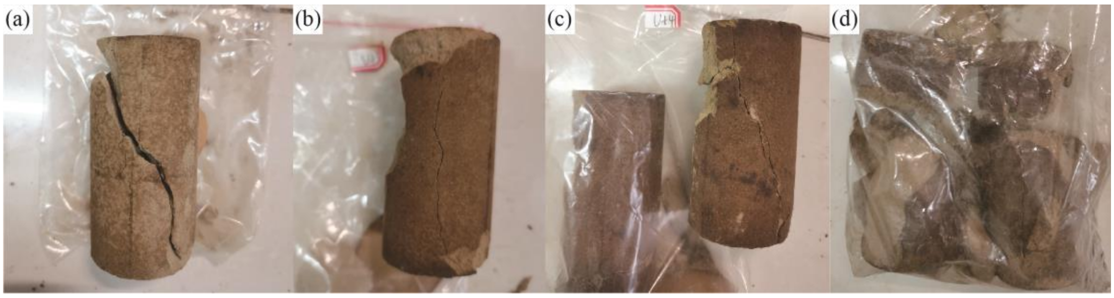

(a) The pressure failure diagram of the constant temperature sample of the E-L scheme

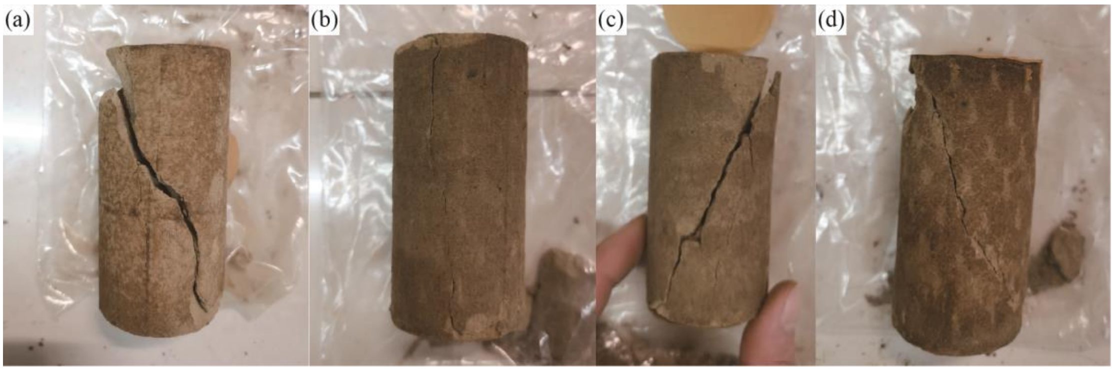

(b) Compression failure diagram of immersed samples in E-L scheme

Figure S6. Compression failure diagram of immersed samples in E-L scheme.

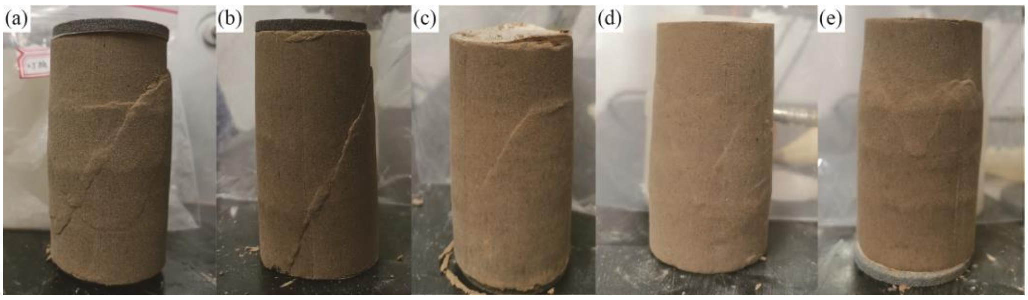

Figure S7. The failure diagram of the sample of scheme E under the action of dynamic load.

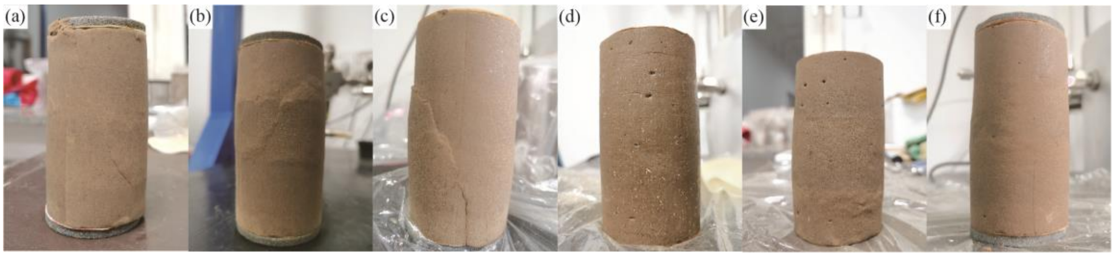

Figure S8 Failure diagram of L scheme specimen under dynamic load

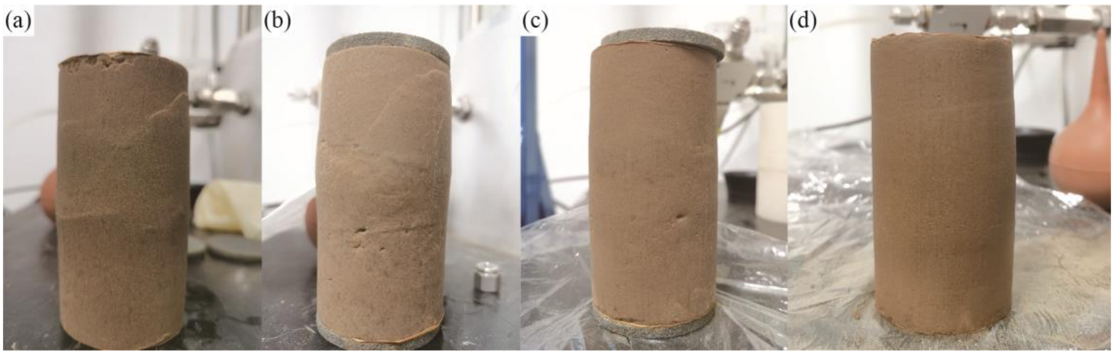

Figure S9. Failure diagram of E-L scheme specimen under dynamic load.
